# Bioethical principles and values during pandemics

**DOI:** 10.6061/clinics/2020/e2154

**Published:** 2020-08-14

**Authors:** Chin An Lin, Henrique Grunspun, Janice Caron Nazareth, Reinaldo Ayer de Oliveira

**Affiliations:** I Medicina Interna Geral, Hospital das Clinicas HCFMUSP, Faculdade de Medicina, Universidade de Sao Paulo, Sao Paulo, SP, BR.; II Comite de Bioetica, Hospital das Clinicas HCFMUSP, Faculdade de Medicina, Universidade de Sao Paulo, Sao Paulo, SP, BR.; III Hospital Israelita Albert Einstein, Sao Paulo, SP, BR.; IV Hospital Alemao Oswaldo Cruz, Sao Paulo, SP, BR.; V Disciplina de Bioetica, Faculdade de Medicina FMUSP, Universidade de Sao Paulo, Sao Paulo, SP, BR.

## INTRODUCTION

The coronavirus disease 2019 (COVID-19) pandemic is the greatest threat to public health today. The disease arose at a time when it was widely believed that humankind had only one last frontier to conquer in public health—to master techniques for the definitive treatment of genetic and hereditary diseases, as well as neoplasms and degenerative diseases. The emergence of a new infectious disease, caused by a virus previously believed to be harmless to human beings, generated a global crisis and caused the medical scientific community to thoroughly review existing concepts concerning prevention, epidemiology, and treatment (1).

The crisis caused by COVID-19 not only affected individuals’ health, but also exposed a need for reflection on social and cultural habits, the means of economic production, public administration policies, and government functioning, amongst other important issues (2,3,4). It highlighted a crisis of values, demanding a review of medical activity priorities, research investments, and financing in health science, as well as the gap between individual and collective health. In addition to compromising health and threatening survival at both the individual and collective levels, this crisis raised questions about our current approaches to the environment and sustainability.

We are being challenged and surrounded by uncertainties, and simultaneously learning how to deal with the pandemic and its sudden, merciless, and guideless outbreak. The learning opportunity has been immense, but the scientific community has been reduced to mere observers - as if we are enrolled in an observational-type cohort study - in which exposure to the virus leads to the first set of observed results. Major epidemics such as COVID-19, natural disasters that threaten large populations, and climatic emergencies and their consequences, demand effective management and public health actions, as well as environmental, socio-economic, and health policies. These areas, however, lack comprehensive or cohesive bioethical consideration.

The principles of bioethics are indispensable across all levels of coping with the pandemic, ranging from patient care to the issue of resource allocation. A theoretical framework is required to establish and support decisions, which, although legal, lack an ethical basis to be clearly translated to affected individuals and populations.

### Beneficence and non-maleficence

Ensuring adequate levels of public health care clearly constitutes the cornerstone of the global fight against the pandemic, driving all aspects of COVID-19 therapy. The principles of beneficence and non-maleficence guide all parameters of patient care. Thus, any decision—even those based on epidemiological and scientific evidence—may appear like mere authoritarian impositions if they are not perceived through an ethical lens (5).

Thus, health professionals should adopt the perspective of collectivity—and not individual or specific measures of care—when discussing the evidence regarding the effectiveness of measures, whether therapeutic, device-based, or behavioral. Public comments made without solid scientific evidence could exacerbate health problems and generate conflicts. Thus, it is mandatory for professionals to exercise extra care in their discourse so as not to expose information that could bring potential harm to the population (6).

In the current scenario, discussing information without appropriate evidence can lead to political antagonism, contaminate the medical and scientific environment, and lead to disputes that are not grounded on science, but rather, on political passions, which certainly does not contribute to the non-maleficence principle. The preservation of these principles is necessarily based on the veracity of information regarding the diagnosis, risks of treatment courses, adverse effects of medication, and scientific research protocol involving human beings, on which bioethics is founded.

### Equity and autonomy

The above-mentioned topics are especially significant in bioethics and require in-depth discussions. Moreover, the long durations for which intensive care beds are occupied, and the lack of human and material resources to meet the pandemic’s needs, demand swift and extensive discussions on resource allocation, as well as the exercise of equity and autonomy. Should human dignity be reduced to the values on some cold score scales, with patient's probability of survival, quality of life after recovery and age being parameters when deciding upon the allocation of intensive care beds? (7)

In the context of a pandemic, clinical severity criteria should not be considered in isolation. The probability of survival, years gained and quality of life after intensive care, and previous existing comorbidities should also be assessed as part of the criteria for admission to intensive care units. With the outbreak of COVID-19, care of patients with important chronical diseases like diabetes, metabolic syndromes, arterial hypertension, chronic obstructive pulmonary disease, dyslipidemia, cardiovascular diseases, and neoplasms was postponed, with allocation of main resources to face the pandemic, remaining but little attention towards these patients. The huge burden will arise when these patients worsen because of lack of care, bringing up an after pandemic crisis in public health. This scenario invites us to a deeper reflection, concerning to prioritization policies on public health (8).

### Challenges in palliative care during the pandemic

Care that is proportional to the expectation of survival must be guaranteed to everyone, to reduce the suffering of both patients and their families. This has in turn necessitated greater discussion around the use of technology to supplement human care. The application of technology is not simply a temporary solution to the pandemic, but has potential for wider use in the future in areas such as end-of-life care, as well as in response to issues including refusal of treatment, shared decisions, conscientious objection, and advance directive of will.

The principle of autonomy, in relation to both the patient and the health care team, must be respected. This equity can only be achieved through effective communication, involving detailed analysis of the pros and cons of each option, to provide the patient and the patient’s representatives with the necessary basis for decision-making as well as deep and reciprocal respect, to reach a true shared decision (9).

### Remote assistance

Due to the high risk of contagion, isolation and confinement measures adopted during the pandemic had a major impact on care and family relationships. Telemedicine was adopted on a large scale, despite a lack of adequate discussion regarding its formalization and adoption. Due to isolation and social distance measures, many professional orientations had to be performed using technology, through electronic devices and applications with an internet connection. The need to maintain professional secrecy, medical records, and confidentiality in these teleconsultations requires an improvement to existing systems, as they are not immune to cyber-attacks. Thus, these topics need to be considered in the post-pandemic period (10).

Furthermore, the impossibility to authorize procedures and diagnostic tests, and consult with seriously ill patients it is necessary to have videoconferences systems in place. Such videoconferences can help doctors obtain consent for procedures from patients’ legal representatives, or even virtually consult with and monitor patients. Certainly, these unprecedented ways to receive authorization or refusal, have become as valid as physical signatures once they are registered in the medical record itself. Videoconferences with families are an excellent means of providing assistance and dignity to in-patients, and thus, should be maintained as auxiliary instruments in the future (11).

## CONCLUSION

During calamities, individual survival depends on collective survival, and individual protective actions depend on synergistic collective action. No individual can protect only himself or herself during a pandemic or natural disaster. Every protective or preventive action demands a collective approach, even if it curtails individual freedom to a certain degree, especially if such liberty poses a threat to collective well-being.

In addition to the principles, several particular values should be more explicit in reflection and bioethical actions.

The bioethical practice is essentially democratic, seeking to give voice to patients as well as their family members or representatives, in search of their individual value. However, it is also pluralist, in which discussions regarding ethical issues and cases and their dilemmas takes place in multi-disciplinary committees, in which we consider different opinions, even if they are antagonistic.

Another essential value in practice and bioethical action is veracity, as sharing true information regarding the diagnosis, the risks of treatment, the adverse effects of a medicine, and the protocol of scientific research involving human beings, is the foundation on which bioethics is built.

A third fundamental value is solidarity, under which people are not only obliged to each other but to the collective as well—it is a mutual connection between two or more people dependent on each other. Solidarity is an essential and indispensable value in combating a pandemic such as COVID-19. Without solidarity, any public or collective health action is bound to fail.

A fourth value, which concerns the majority of bioethical actions at the individual or collective level, is cooperation. This is understood as acting together with others toward the same purpose.

Without clear ethical values such as democracy, the veracity of information, solidarity among people, and cooperation in collective actions, which can be easily understood by individuals and the community, many of the necessary measures to combat a severe pandemic such as COVID-19 will neither be well accepted nor effective.

The COVID-19 pandemic has led to important reflections, meetings, and discussions, thereby triggering several provocations, actions, and improvements in bioethics committees. This allows not only inter-disciplinary growth but also mediation with health professionals, and enables the population to reconcile and guide different, even antagonistic, opinions. The bioethical reflections resulting from the pandemic will certainly help us emerge better and stronger from the crisis.
